# Association between eye disorders and the development of ADHD/ADD: a nationwide retrospective cohort study

**DOI:** 10.1038/s41433-025-04227-w

**Published:** 2026-01-09

**Authors:** Asaf Israeli, Eedy Mezer

**Affiliations:** 1 Department of Ophthalmology, Vanderbilt Eye Institute, Vanderbilt University Medical Center, Nashville, TN USA; 2 The Ruth and Bruce Rappaport Faculty of Medicine, Technion-Israel Institute of Technology, Haifa, Israel; 3 Department of Ophthalmology, Rambam Health Care Campus, Haifa, Israel; 4 Maccabi Healthcare Services, Haifa, Israel

**Keywords:** Risk factors, Neurological disorders

## Abstract

**Objectives:**

To study and assess the association of various eye disorders with the development of attention-deficit hyperactivity disorder (ADHD/ADD).

**Methods:**

A nationwide, retrospective cohort-study, utilising anonymised electronic medical records (EMR) data on all insured individuals aged 5–30 in Maccabi Health Services (MHS), the second largest health maintenance organisation in Israel, during 2010–2022.

**Results:**

The final analysis included 665,121 individuals from an initial cohort of 1,686,128 after applying selection criteria and propensity score matching. Of these, 68,976 (10.4%) developed ADHD/ADD. ADHD/ADD was more common and developed faster in those with eye disorders (HR = 1.40, 95% CI: 1.38–1.42 and 4.5 versus 4.9 years, *p* < 0.001, respectively). All evaluated eye disorders served as significant risk factors (strabismus: HR = 1.64, 95% CI: 1.49–1.80; hyperopia: HR = 1.52, 95% CI: 1.47–1.56; astigmatism: HR = 1.52, 95% CI: 1.48–1.56; amblyopia: HR = 1.40, 95% CI: 1.27–1.54; myopia: HR = 1.30, 95% CI: 1.28–1.33). Similar findings were evident when assessing combinations of eye disorders. These associations were far more pronounced in females and the paediatric population (*p* < 0.001 and *p* = 0.044, respectively).

**Conclusions:**

Eye disorders serve as risk factors for the development of ADHD/ADD, particularly in females and in the paediatric population.

## Introduction

Global prevalence of attention deficit hyperactivity disorder (ADHD/ADD) is reported to be 2–7%, with an average of 5%. It varies substantially between different countries and is on the rise. For example, in the United States, approximately 27% of children aged 6–17 are being diagnosed with ADHD/ADD, in the UK, children aged 5–15 have a reported prevalence of 3.62% and in Israel, the prevalence is 7.35–9.5% in children aged 7–9 and 3% in adolescents aged 14–17. ADHD/ADD is mostly a male-dominant diagnosis, a phenomenon seen in Israel as well as in other countries [1–6].

Refractive errors are also extremely common worldwide and vary in different countries. The estimated global prevalence of myopia, hyperopia and astigmatism in children is high (11.7, 4.6 and 14.9%, respectively) and even higher in adults (26.5, 30.9 and 40.4%, respectively) [7]. The global prevalence of strabismus is estimated to be 1.93% and that of amblyopia is 1.36% [8, 9]. In Israel, myopia showed a recent increase from 20.4% to 26.2% over a generation [10, 11], while astigmatism and hyperopia in Israel are estimated to be 7.13 and 0.93%, respectively [12]. Strabismus and amblyopia, in contrast, have decreased over recent decades from 1.2 to 0.6% and from 1.2 to 0.8%, respectively. This decrease was attributed to widespread national screening efforts as well as early interventions, which have also aided in decreasing consequential blindness [13–15].

Significant visual impairment can impair a child’s concentration and attentiveness at school. Previous studies have implied a possible connection between eye disorders and ADHD/ADD. A cross-sectional study of 75,000 children utilising parent interviews showed a higher prevalence of ADHD/ADD among individuals with vision problems, yet recall bias, publication bias, snapshot bias and the inability to determine causality may limit the applicability of these results [16].

The goal of this study was to conduct a comprehensive nationwide cohort study to assess the association between eye disorders and the incidence of ADHD/ADD.

## Materials and methods

This retrospective cohort study utilised Electronic Medical Records (EMR) data from Maccabi Health Services (MHS) and encompassed 13 years (2010–2022). Demographic variables included sex, age at eye diagnosis, age at ADHD/ADD diagnosis and ethnicity. Types of eye disorders included refractive errors (i.e. myopia, hyperopia, astigmatism), strabismus (i.e. esotropia, exotropia, hypertropia, hypotropia) and amblyopia (Supplementary Table 1).

### Data collection and sample size

MHS has maintained a continuous nationwide EMR database for over 20 years and is the second largest Health Maintenance Organization (HMO) in Israel, covering over 2.5 million members. The data was directly retrieved from MHS to create an anonymised encoded database. This study included all insured patients in MHS between the ages of 5 and 30. Participants lacking a documented eye examination, rare comorbidities, conditions limiting exam cooperation, diagnoses requiring frequent inpatient follow-ups, or diagnoses often associated with broader systemic or neurological involvement that limit isolating the direct impact of vision (e.g. chiasm disorders, CNS benign and malignant tumours, CNS involvement in systemic diseases, CVA, giant cell arteritis, inflammation, ocular trauma, optic neuritis, optic neuropathies, pathologies involving visual pathways, etc.) were excluded. This was done to avoid additional confounding factors and heterogeneity. The full list of excluded diagnoses alongside their ICD-9 and Y codes (MHS-specific diagnostic codes) is shown in Supplementary Table 2 and the study design is shown in Supplementary Fig. 1.

From an initial cohort of 1,686,128 of all MHS members insured during the study period (2010–2020), we implemented inclusion and exclusion criteria, propensity score matching (PSM) and a wash-out period of at least one year between the index date (i.e. the date of the first eye diagnosis) and the diagnosis of ADHD/ADD. In the final analysis, 221,707 cases were matched to individuals without any eye disorders using a PSM based on sex and birth year at a 1:2 ratio, creating a final cohort of 665,121 participants. The washout period was implemented twice, pre- and post-matching, first to establish the temporal relationship as part of the initial research group creation and then to ensure that the matched controls also do not have an ADHD/ADD diagnosis before the index date of their corresponding research group member. To increase internal validity, only those diagnosed within one year of the index date were included when assessing combinations of eye diagnoses.

### Exposures, outcomes and model covariates

The exposure was defined as having either of the following diagnoses: refractive errors (myopia, hyperopia, astigmatism), strabismus (esotropia, exotropia, hypertropia, hypotropia) and amblyopia. The term ‘General eye diagnosis’ indicates having at least one of these diagnoses. The outcome was defined as the diagnosis of ADHD/ADD. Age and sex were accounted for through PSM. The reference group for all comparisons consisted of matched individuals without any eye diagnoses (‘Without eye diagnosis’).

### Disorder classification and diagnostic standards

The diagnostic criteria for all eye disorders were applied uniformly across the study population. Specifically, refractive errors such as myopia, hyperopia and astigmatism were based on standardised refractive assessments. The common practice in Israel is to perform manifest refraction using a phoropter in adults and an age-adjusted cycloplegic retinoscopy in children in order to neutralise accommodation and ensure accurate refractive measurement. Strabismus was diagnosed through clinical evaluation of eye misalignment and amblyopia was identified by best-corrected visual acuity not explained by structural abnormalities. All diagnoses are made by board-certified ophthalmologists or optometrists following established clinical guidelines. ADHD/ADD diagnoses are made exclusively by board-certified paediatric psychiatrists or developmental paediatric neurologists, generally following DSM-5 criteria, reflecting standard clinical practice and ensuring consistency across the cohort.

### Ethical issues

Patients’ information was collected anonymously and encoded to ensure anonymity using MDClone software (MDClone®, MDClone Ltd., Be’er Sheva, Israel). The study did not require informed consent and an Institutional Review Board (IRB) approval was obtained from the MHS IRB, Tel-Aviv, Israel (No. MHS 0017-23).

### Statistical analysis

Data were analysed by SPSS Statistical Package (IBM, Version 29, Armonk, NY, USA). Continuous variables were reported as mean ± SD or median [Q1–Q3], as appropriate. Differences between groups were tested using a t-test or a Mann-Whitney test, as appropriate. Categorical data were reported as frequency and percentage and associations were tested using the Chi-square test. Cox regression analysis was used to assess the hazard ratios (HR) and 95% confidence intervals (CI) of ADHD/ADD risk associated with eye diagnoses. Analyses for general eye diagnosis were repeated while stratifying for age and sex and using a test for heterogeneity in order to compare between groups. *P*-values of 5% were considered statistically significant.

## Results

665,121 patients were evaluated in this study after inclusion and exclusion criteria and after the matching process were implemented (221,707 and 443,414, with and without an eye disorder, respectively). Of the total sample, 68,976 individuals (10.4%) were diagnosed with ADHD/ADD. This included 27,948 (12.6%) among those with an eye disorder, compared to 41,028 (9.3%) among those without an eye disorder (*p* < 0.001). The mean age was 14.8 ± 7.5 years and 41.1% were male (Table 1).

**Table 1:** Demographics, overall and by the presence of an eye disorder.

	Total *N* = 665,121	Without eye diagnosis *N* = 443,414	With eye diagnosis *N* = 221,707	*p*-value
Age, mean ± SD	14.8 ± 7.5	14.8 ± 7.5	14.8 ± 7.5	1.000
Sex (male), *n* (%)	273,255 (41.1)	182,170 (41.1)	91,085 (41.1)	1.000
Country birth (Israel), *n* (%)	610,020 (91.7)	401,831 (90.6)	208,188 (93.9)	<0.001
Ethnicity (Jews), *n* (%)	601,761 (90.5)	392,511 (88.8)	209,250 (94.5)	<0.001
Socioeconomic Status				
1–4	181,489 (27.3)	119,710 (27.0)	61,779 (27.9)	<0.001
5–7	277,603 (41.8)	187,567 (42.4)	90,036 (40.7)	
8–10	204,990 (30.9)	135,323 (30.6)	69,667 (31.5)	
ADHD/ADD	68,976 (10.4)	41,028 (9.3)	27,948 (12.6)	<0.001
Follow-up time in years, mean ± SD	7.7 ± 3.6	7.8 ± 3.6	7.6 ± 3.7	<0.001

Overall, having an eye disorder was associated with ADHD/ADD (HR = 1.40, *p* < 0.001). Similarly strong association results were obtained regardless of either medication prescription or medication dispensation (HR = 1.41, *p* < 0.001 and HR = 1.42, *p* < 0.001, respectively) (Supplementary Table 3). All evaluated eye diagnoses served as significant risk factors (strabismus: HR = 1.64, 95% CI: 1.49–1.80; hyperopia: HR = 1.52, 95% CI: 1.47–1.56; astigmatism: HR = 1.52, 95% CI: 1.48–1.56; amblyopia: HR = 1.40, 95% CI: 1.27–1.54; myopia: HR = 1.30, 95% CI: 1.28–1.33) (Table 2). Socioeconomic status differences were statistically significant but clinically insignificant. To validate this, a covariate sensitivity analysis was employed and showed no effect on the results (Supplementary Table 4).

**Table 2:** Associations between eye disorders and ADHD/ADD.

	No ADHD/ADD		ADHD/ADD		HR [95% CI]	*p*-value
	*N*	%	*N*	%		
**General eye diagnosis**						
Without eye diagnosis (*n* = 443,414)	402,386	67.5	41,028	59.5	1.40 [1.38–1.42]	<0.001
With the diagnosis (*n* = 221,707)	193,759	32.5	27,948	40.5		
**Strabismus**						
Without eye diagnosis (*n* = 8950)	8008	68.1	942	56.3	1.64 [1.49-1.80]	<0.001
With the diagnosis (*n* = 4475)	3743	31.9	732	43.7		
**Amblyopia**						
Without eye diagnosis (*n* = 9560)	8508	67.7	1052	59.6	1.40 [1.27–1.54]	<0.001
With the diagnosis (*n* = 4780)	4068	32.3	712	40.4		
**Myopia**						
Without eye diagnosis (*n* = 286,282)	262,582	67.2	23,700	61.0	1.30 [1.28–1.33]	<0.001
With the diagnosis (*n* = 143,141)	127,966	32.8	15,175	39.0		
**Hyperopia**						
Without eye diagnosis (*n* = 81,210)	71,179	68.1	10,031	57.9	1.52 [1.47–1.56]	<0.001
With the diagnosis (*n* = 40,605)	33,313	31.9	7292	42.1		
**Astigmatism**						
Without eye diagnosis (*n* = 118,814)	106,823	67.8	11,991	57.7	1.52 [1.48–1.56]	<0.001
With the diagnosis (*n* = 59,407)	50,631	32.2	8776	42.3		

^*^n represents the number of matched cases and controls (in a 1:2 ratio) with the eye diagnosis in question and without an eye diagnosis, respectively.

**N represents the number of participants in each cell defined by ADHD/ADD status and eye diagnosis category; % indicates the proportion within each ADHD/ADD group.

**Fig. 1:**
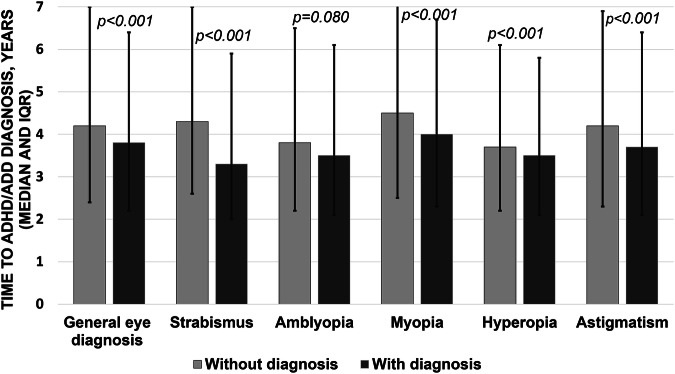
Comparison of time to ADHD/ADD diagnosis with and without an eye disorder.

The mean time to develop ADHD/ADD was significantly shorter in individuals diagnosed with an eye disorder (4.5 versus 4.9 years, *p* < 0.001). This pattern was evident across all diagnoses evaluated (*p* < 0.001), except for amblyopia, possibly due to a smaller sample size (4.3 versus 4.6 years, *p* = 0.080) (Fig. 1 and Supplementary Table 5).

Subsequently, several combinations of eye disorders were evaluated as well. The strongest significant association was strabismus, hyperopia and astigmatism (HR = 1.71, *p* = 0.043), followed by strabismus and hyperopia (HR = 1.56, *p* < 0.001), hyperopia and astigmatism (HR = 1.53, *p* < 0.001) and strabismus and myopia (HR = 1.49, *p* < 0.001). Some combinations showed a strong association but did not reach statistical significance, most likely due to smaller sample sizes (e.g. strabismus, amblyopia and myopia with an HR = 2.72, *p* = 0.135) (Table 3).

**Table 3:** Associations between combinations of eye disorders and ADHD/ADD.

	No ADHD/ADD		ADHD/ADD		HR [95% CI]	*p*-value
	*N*	%	*N*	%		
**Strabismus and amblyopia**						
Without eye diagnosis (*n* = 250)	216	67.9	34	59.6	1.41 [0.83–2.39]	0.283
With the diagnosis (*n* = 125)	102	32.1	23	40.4		
**Strabismus and myopia**						
Without eye diagnosis (*n* = 1516)	1,370	67.7	146	58.2	1.49 [1.16–1.91]	0.002
With the diagnosis (*n* = 758)	653	32.3	105	41.8		
**Strabismus and hyperopia**						
Without eye diagnosis (*n* = 1346)	1178	68.3	168	57.3	1.56 [1.24–1.96]	<0.001
With the diagnosis (*n* = 673)	548	31.7	125	42.7		
**Strabismus and astigmatism**						
Without eye diagnosis (*n* = 1036)	920	67.8	116	58.9	1.46 [1.10–1.94]	0.009
With the diagnosis (*n* = 518)	437	32.2	81	41.1		
**Amblyopia and myopia**						
Without eye diagnosis (*n* = 1254)	1,138	67.5	116	59.5	1.40 [1.05–1.86]	0.022
With the diagnosis (*n* = 627)	548	32.5	79	40.5		
**Amblyopia and hyperopia**						
Without eye diagnosis (*n* = 3042)	2619	67.3	423	62.8	1.22 [1.04–1.4542	0.015
With the diagnosis (*n* = 1521)	1,270	32.7	251	37.2		
**Amblyopia and astigmatism**						
Without eye diagnosis (*n* = 2640)	2264	67.7	376	60.9	1.33 [1.13–1.56]	0.001
With the diagnosis (*n* = 1320)	1079	32.3	241	39.1		
**Myopia and astigmatism**						
Without eye diagnosis (n = 40,806)	37,335	67.5	3581	59.0	1.43 [1.36–1.50]	<0.001
With the diagnosis (*n* = 20,403)	17,913	32.5	2490	41.0		
**Hyperopia and astigmatism**						
Without eye diagnosis (*n* = 19,072)	16,572	68.2	2500	57.8	1.53 [1.44–1.63]	<0.001
With the diagnosis (*n* = 9536)	7710	31.8	1826	42.2		
**Strabismus, amblyopia and myopia**						
Without eye diagnosis (*n* = 36)	32	71.1	4	44.4	2.72 [0.73–10.15]	0.135
With the diagnosis (*n* = 18)	13	28.9	5	55.6		
**Strabismus, amblyopia and hyperopia**						
Without eye diagnosis (*n* = 98)	85	66.4	13	68.4	0.91 [0.34–2.38]	0.840
With the diagnosis (*n* = 49)	43	33.6	6	31.6		
**Strabismus, amblyopia and astigmatism**						
Without eye diagnosis (*n* = 56)	47	65.3	9	75.0	0.67 [0.18–2.49]	0.554
With the diagnosis (*n* = 28)	25	34.7	3	25.0		
**Strabismus, hyperopia and astigmatism**						
Without eye diagnosis (*n* = 288)	256	68.4	32	55.2	1.71 [1.02–2.87]	0.043
With the diagnosis (*n* = 144)	118	31.6	26	44.8		
**Strabismus, amblyopia, myopia and astigmatism**						
Without eye diagnosis (*n* = 14)	12	70.6	2	50.0	2.41 [0.34–17.21]	0.379
With the diagnosis (*n* = 7)	5	29.4	2	50.0		
**Strabismus, amblyopia, hyperopia and astigmatism**						
Without eye diagnosis (*n* = 26)	24	66.7	2	66.7	0.97 [0.09–10.72]	0.981
With the diagnosis (*n* = 13)	12	33.3	1	33.3		

^*^n represents the number of matched cases and controls (in a 1:2 ratio) with the eye diagnosis in question and without an eye diagnosis, respectively.

**N represents the number of participants in each cell defined by ADHD/ADD status and eye diagnosis category; % indicates the proportion within each ADHD/ADD group.

**Fig. 2:**
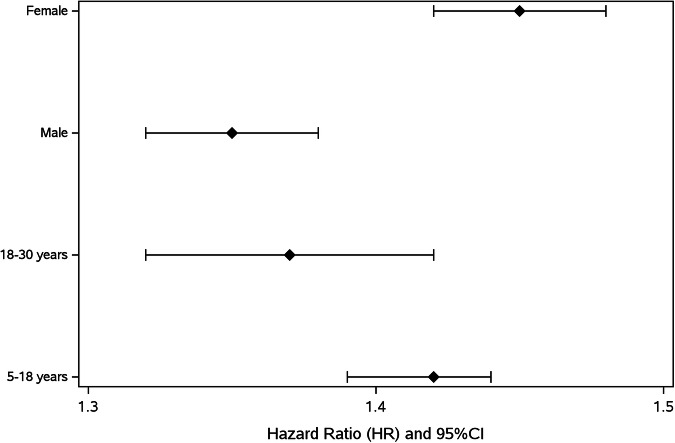
Associations between eye disorders and ADHD/ADD, by age group and sex.

Lastly, sex- and age-disparities were evaluated with regard to the association between eye diagnoses and ADHD/ADD through subgroup analyses—using stratification and test for heterogeneity. This association was notably stronger in females compared to males (*p* < 0.001) and in the paediatric population compared to adults (*p* = 0.044) (Fig. 2 and Supplementary Table 6).

## Discussion

In this comprehensive, nationwide, retrospective cohort study of 665,121 patients drawn from an initial cohort of 1,686,128 over a 13-year period, we found a higher incidence of ADHD/ADD among patients with eye disorders—namely refractive errors, strabismus and amblyopia compared to those without any eye disorders. ADHD/ADD severity verified by proof of a prescription on file, or documented medication dispensation, did not affect this association. All these eye diagnoses showed a strong association and served as significant risk factors.

Previous studies from Taiwan, Germany and Korea have shown that children afflicted with ADHD/ADD have a higher prevalence of vision impairment due to eye disorders [17–19] and children with significant eye disorders are more afflicted with ADHD/ADD than other children in Korea [20]. Two reports from Israel, both from a single paediatric ophthalmology clinic, have shown conflicting results. One showed a 20% prevalence of significant hyperopia in children with ADHD/ADD, while the other reported no association of ADHD/ADD with refractive errors; however, selection bias and small sample sizes decrease the generalisation of the results in these 2 studies [21, 22].

ADHD/ADD was also found to develop significantly earlier in patients with any of the aforementioned eye disorders, by 4.8 months (45.6 vs. 50.4 months), compared to those without eye disorders. This was observed for individuals with refractive errors or strabismus, but not for those with amblyopia, possibly due to a smaller sample size. These findings could potentially indicate overlapping neurodevelopmental pathways, shared genetic culprits or, conversely, simply ascertainment bias [23]. It is possible that early neurodevelopmental screening would be beneficial in children with eye disorders, but the clinical significance is yet to be determined at this point.

Several combinations of eye disorders were also found to be significantly associated with ADHD/ADD, most notably strabismus, hyperopia and astigmatism and pairings of these combinations’ components (strabismus and hyperopia, as well as astigmatism and hyperopia).

Interestingly, combinations of these diagnoses were significantly associated with ADHD/ADD as well; however, no clear synergistic effects were observed. A possible explanation could be that the association may derive from the direct impact of visual impairment itself, rather than distinct underlying pathological mechanisms.

Various eye disorders and ADHD/ADD are both principally related to and affected by sex and age. These differences could be related to different pathophysiology or biases, such as referral bias [11, 14, 15, 24–27]. In this cohort, the association between eye disorders and ADHD/ADD was substantially more significant in females as opposed to males and far more pronounced in children and teenagers compared to adults. This could potentially be related to biological, physiological, or socio-environmental factors, as well as to differences in eye disorder types, with some tending to present and/or be screened for in an earlier age, or disease severity prompting sooner evaluation by an ophthalmologist. Another possible explanation could stem from higher rates of underdiagnosis of ADHD/ADD in the adult population [28].

While vision disorders could cause ADHD/ADD symptoms, conversely, it is also essential to consider that hyperactivity and lack of concentration during various tasks, including visual acuity evaluations, might also masquerade as eye problems [29, 30]. These may pose possible biases and erroneous diagnoses of children, as well as cause a delay in treatment or lead to improper treatment.

Notably, unlike strabismus and amblyopia, refractive error is easily correctable and often underdiagnosed. This could potentially decrease the effect size of our results. However, this is minimised by several factors: Israel’s routine state-wide mandatory ophthalmological assessments at young ages and during military conscription processes, free universal healthcare with comprehensive documentation in Israeli HMOs and the age limitations for this study [11, 14, 15, 31].

In that regard, it is also pertinent to distinguish between correctable pathologies and permanent ones, as these may have different effects. Albeit beyond the scope of this study, future studies investigating this distinction further could better elucidate the impact of these differences.

This study has several limitations. Firstly, its retrospective nature may obscure confounding variables and limit the ability to establish a temporal relationship. However, this was mitigated by matching for age and sex, as well as incorporating a one-year washout period to strengthen validity. Another limitation is its reliance on EMR, which is contingent upon physicians’ compliance and accuracy. Lastly, educational level and access to healthcare could not be accounted for due to their absence from electronic records. That said, Israel’s free universal healthcare system, small geographic size and the legal obligation of all its residents to have HMO memberships—all mitigate proper access to medicine, diminish this limitation and contribute to generalisability [31, 32]. This study also has other strengths, such as the nationwide comprehensive database of the 2nd largest HMO in the country, the uniformity of data, the very large sample size, as well as previously reported similarities to other Western countries [14, 15, 33].

In conclusion, refractive disorders, strabismus and amblyopia are significantly associated with ADHD/ADD, particularly in children and teenagers. This association is also far more pronounced in females. These findings can set up a detailed framework that may aid primary care providers and neurologists with risk stratification and early detection and intervention of ADHD/ADD, as well as guide ophthalmologists to refer high-risk patients for proper evaluation. Additionally, these associations could prompt future research into specific conditions and their association with ADHD/ADD.

### Disclaimer

The authors take responsibility for all aspects of reliability and freedom from bias of the data presented and their discussed interpretation.

## Summary

### What is known about this topic

- Previous studies reported a higher prevalence of eye disorders among children diagnosed with ADHD/ADD.
- However, these studies were mostly cross-sectional, involved small sample sizes and did not explore reverse associations or disparities by age or sex.

### What this study adds

- Eye disorders serve as risk factors for the development of ADHD/ADD.
- These associations are particularly evident in females, as well as in the paediatric population, compared to adults.
- Primary care providers and neurologists may use the presented risk stratification for early screening and intervention of ADHD/ADD. Ophthalmologists should refer high-risk patients for proper evaluation.

## Supplementary information

Supplementary Figure 1

Supplementary Table 1

Supplementary Table 2

Supplementary Table 3

Supplementary Table 4

Supplementary Table 5

Supplementary Table 6

## Data Availability

Data sharing will not be permitted due to confidentiality constraints from the Israeli Ministry of Health.
