# Supplementary material for: Association between eye disorders and the development of ADHD/ADD: a nationwide retrospective cohort study
Source: Eye (Lond). 2026 Jan 9;40(4):550–6. doi: 10.1038/s41433-025-04227-w (PMC12957306; doi:10.1038/s41433-025-04227-w)
Supplement: Supplementary file 1 — Supplementary Figure 1 [file 41433_2025_4227_MOESM1_ESM.docx]

MHS members 2010-2022

N=1,686,128

Members without eye disorders
 N=1,104,206

Members with eye disorders
 N=581,922

Exclusion criteria
 N=4,350

Exclusion criteria
 N=6,578

ADHD/ADD diagnosis not during 2010-2022
 N=41,411

ADHD/ADD diagnosis not during 2010-2022

N=51,593

Eye diagnosis not during 2010-2022
 N=106,902

Washout period - excluding cases in which ADHD/ADD was diagnosed less than one year after an eye diagnosis was made N=44,552

Eye diagnosis not at age 5-30

N=101,042

Included members without eye disorders
N=1,048,263

Included members with eye disorders
N=281,437

Matching 1:2 by sex and year of birth and excluding participants with ADHD/ADD diagnosis less than one year after index date (eye diagnosis)

Included members with eye disorders
N=221,707

Included members without eye disorders
N=443,414
