# Supplementary material for: Association between eye disorders and the development of ADHD/ADD: a nationwide retrospective cohort study
Source: Eye (Lond). 2026 Jan 9;40(4):550–6. doi: 10.1038/s41433-025-04227-w (PMC12957306; doi:10.1038/s41433-025-04227-w)
Supplement: Supplementary file 2 — Supplementary Table 1 [file 41433_2025_4227_MOESM2_ESM.docx]

| **Eye diagnosis** | **ICD9 and Y codes** |
| --- | --- |
| Refractive error | Y13164 |
| Myopia | Y13151, 367.1 |
| Progressive high (degenerative) myopia malignant myopia | Y12807, 360.21 |
| Hyperopia | Y13148, 367 |
| Astigmatism | Y13154, 367.2 |
| Strabismus uns | Y13656, 378.9 |
| Strabismus mechanical uns | Y13639, 378.6 |
| Strabismus paralytic | Y13634, 378.5 |
| Strabismus mechanical musculofacial dis | Y13638, 378.62 |
| Limited duction mechanical strabismus | Y13641, 378.63 |
| Strabismus neuromuscular dis uns | Y13642, 378.73 |
| Strabismus surgery | Y23111, V45.69 |
| Deprivation amblyopia | Y13168, 368.02 |
| Suppression amblyopia | Y13167 |
| Amblyopia uns | Y13165, 368 |
| Refractive amblyopia | Y13171, 368.03 |
| Strabismus amblyopia strabismic | Y13166 |
| Esotropia | Y13590, 378 |
| Exotropia | Y13600, 378.1 |
| Hypertropia | Y13617, 378.31 |
| Hypotropia | Y13618, 378.32 |

Supplemental Table 1. ICD-9 and Y codes of included eye diagnoses.

*Y codes are MHS-specific diagnostic codes.
