# Supplementary material for: Association between eye disorders and the development of ADHD/ADD: a nationwide retrospective cohort study
Source: Eye (Lond). 2026 Jan 9;40(4):550–6. doi: 10.1038/s41433-025-04227-w (PMC12957306; doi:10.1038/s41433-025-04227-w)
Supplement: Supplementary file 3 — Supplementary Table 2 [file 41433_2025_4227_MOESM3_ESM.docx]

Supplemental Table 2. ICD-9 and Y codes for excluded diagnoses.

| **Exclusion criteria** | **ICD9 / Y CODE** |
| --- | --- |
| HEMORRHAGIC CVA | Y14232 |
| HEMORRHAGIC CVA | 431 |
| EMBOLISM CEREBRAL | Y14245 |
| OPTIC CHIASM DISS NEOPLASTIC NOT PITUIT | Y13578 |
| VISUAL PATHWAY NEOPLASM | Y13580 |
| VISUAL PATHWAY NEOPLASM | 377.61 |
| VISUAL CORTEX NEOPLASM | Y13584 |
| VISUAL CORTEX NEOPLASM | 377.71 |
| MALIGNANT NEOPLASM OF BRAIN | Y20429 |
| NUTRITIONAL OPTIC NEUROPATHY | 377.33 |
| MALIGNANT NEOPLASM OF EYE | 190 |
| MALIGNANT NEOPLASM OF EYEBALL, EXCEPT CONJUNCTIVA, CORNEA, RETINA, AND CHOROID | 190.0 |
| MALIGNANT NEOPLASM OF ORBIT | 190.1 |
| MALIGNANT NEOPLASM OF LACRIMAL GLAND | 190.2 |
| MALIGNANT NEOPLASM OF CONJUNCTIVA | 190.3 |
| MALIGNANT NEOPLASM OF CORNEA | 190.4 |
| MALIGNANT NEOPLASM OF RETINA | 190.5 |
| MALIGNANT NEOPLASM OF CHOROID | 190.6 |
| MALIGNANT TUMOR LACRIMAL DUCT PRIM | 190.7 |
| MALIGNANT NEOPLASM OF OTHER SPECIFIED SITES OF EYE | 190.8 |
| MALIGNANT NEOPLASM OF EYE, PART UNSPECIFIED | 190.9 |
| EYE CA IN SITU | 234.0 |
| PERSONAL HISTORY OF MALIGNANT NEOPLASM OF EYE | V10.84 |
| ORBIT PRIM MALIGNANT TUMOR | Y10880 |
| RETINOBLASTOMA | Y10881 |
| MALIGNANT TUMOR LACRIMAL DUCT PRIM | Y10882 |
| MALIGNANT TUMOR EYE PRIM | Y10883 |
| EYE METASTASES TO | Y10941 |
| EYE CA IN SITU | Y11343 |
| TUBERCULOSIS EYE | 017.3 |
| TOXIC AMBLYOPIA | Y13570 |
| INFRARED RADIATION INDUCED DISEASE INC. CATARACT | Y21874 |
| MICRO/RADIO WAVE RADIATION DIS INC. CATARACT | Y21876 |
| CATARACT TRAUMATIC | Y13120 |
| TRAUMATIC CATARACT TOTAL | Y13123 |
| TRAUMATIC CATARACT PARTIALLY RESOLVED | Y13124 |
| CATARACT SEC TO GLAUCOMA :(CATARACT SECONDARY TO GLAUCOMA) | Y13127 |
| CATARACT IN INFLAMMATORY DISORDERS | Y13128 |
| CATARACT+NEOVASCULARIZATION | Y13129 |
| SUNFLOWER CATARACT | Y13130 |
| DIABETIC CATARACT | Y13131 |
| TETANIC CATARACT | Y13133 |
| MYOTONIC CATARACT | Y13135 |
| TOXIC CATARACT | Y13137 |
| CATARACT+RADIATION+OTHER PHYSICAL AGENTS | Y13139 |
| CATARACT LASER INDUCED | Y13140 |
| CATARACT RADIATION INDUCED | Y13141 |
| SECONDARY CATARACT | Y13142 |
| CATARACT TRAUMATIC | 366.2 |
| TRAUMATIC CATARACT TOTAL | 366.22 |
| TRAUMATIC CATARACT PARTIALLY RESOLVED | 366.23 |
| CATARACT SECONDARY TO OCULAR DISORDERS | 366.3 |
| CATARACT IN INFLAMMATORY DISORDERS | 366.32 |
| CATARACT+NEOVASCULARIZATION | 366.33 |
| MYOTONIC CATARACT | 366.43 |
| CATARACT+RADIATION+OTHER PHYSICAL AGENTS | 366.46 |
| GLAUCOMATOCYCLITIC CRISES | 364.22 |
| GLAUCOMA BORDERLINE | 365.0 |
| GLAUCOMA OPEN ANGLE | 365.1 |
| PRIMARY OPEN ANGLE GLAUCOMA | 365.11 |
| LOW TENSION GLAUCOMA | 365.12 |
| PIGMENTARY GLAUCOMA | 365.13 |
| RESIDUAL STAGE OPEN ANGLE GLAUCOMA | 365.15 |
| GLAUCOMA PRIMARY ANGLE CLOSURE | 365.2 |
| PRIMARY ANGLE CLOSURE GLAUCOMA UNS | 365.20 |
| INTERMITTENT ANGLE CLOSURE GLAUCOMA | 365.21 |
| GLAUCOMA CHRONIC ANGLE CLOSURE | 365.23 |
| RESIDUAL STAGE ANGLE CLOSURE GLAUCOMA | 365.24 |
| GLAUCOMA CORTICOSTEROID INDUCED | 365.3 |
| GLAUCOMA+CONGENITAL ANOMALIES;DYSTROPHIES;SYSTEMIC SYNDROMES | 365.4 |
| GLAUCOMA DUE TO DISORDERS OF THE LENS | 365.5 |
| PHACOLYTIC GLAUCOMA | 365.51 |
| PSEUDOEXFOLIATION GLAUCOMA | 365.52 |
| GLAUCOMA ASS WITH OTHER OCULAR DISORDERS | 365.6 |
| GLAUCOMA IRIDOCORNEAL ENDOTHELIAL (ICE) SYNDROME | 365.60 |
| GLAUCOMA+PUPILLARY BLOCK | 365.61 |
| GLAUCOMA ASS WITH OCULAR INFLAMMATION | 365.62 |
| GLAUCOMA ASS WITH VASCULAR DISORDERS | 365.63 |
| GLAUCOMA ASS WITH TUMORS OR CYSTS | 365.64 |
| GLAUCOMA+OCULAR TRAUMA | 365.65 |
| HYPERSECRETION GLAUCOMA | 365.81 |
| GLAUCOMA+++ EPISCLERAL VENOUS PRESSURE | 365.82 |
| GLAUCOMA UNS | 365.9 |
| HYDROPHTHALMOS | 743.2 |
| GLAUCOMATOCYCLITIC CRISES | Y13023 |
| GLAUCOMA BORDERLINE | Y13070 |
| GLAUCOMA SUSPECT ANATOMICAL NARROW ANGLE | Y13071 |
| GLAUCOMA SUSPECT OCULAR HYPERTENSION | Y13072 |
| GLAUCOMA SUSPECT OPEN ANGLE BORDER FINDING | Y13073 |
| GLAUCOMA SUSPECT STEROID RESPONDERS | Y13074 |
| GLAUCOMA OPEN ANGLE | Y13075 |
| PIGMENTARY GLAUCOMA | Y13076 |
| PRIMARY OPEN ANGLE GLAUCOMA | Y13077 |
| LOW TENSION GLAUCOMA | Y13078 |
| GLAUCOMA CHILDHOOD | Y13079 |
| RESIDUAL STAGE OPEN ANGLE GLAUCOMA | Y13080 |
| GLAUCOMA PRIMARY ANGLE CLOSURE | Y13081 |
| PRIMARY ANGLE CLOSURE GLAUCOMA UNS | Y13082 |
| INTERMITTENT ANGLE CLOSURE GLAUCOMA | Y13083 |
| GLAUCOMA CHRONIC ANGLE CLOSURE | Y13084 |
| RESIDUAL STAGE ANGLE CLOSURE GLAUCOMA | Y13086 |
| GLAUCOMA CORTICOSTEROID INDUCED | Y13087 |
| GLAUCOMA+CONGENITAL ANOMALIES;DYSTROPHIES;SYSTEMIC SYNDROMES | Y13089 |
| GLAUCOMA DUE TO DISORDERS OF THE LENS | Y13091 |
| PHACOLYTIC GLAUCOMA | Y13093 |
| PSEUDOEXFOLIATION GLAUCOMA | Y13094 |
| GLAUCOMA+PUPILLARY BLOCK | Y13095 |
| GLAUCOMA ASS WITH OTHER OCULAR DISORDERS | Y13096 |
| GLAUCOMA ASS WITH OCULAR INFLAMMATION | Y13097 |
| GLAUCOMA ASS WITH VASCULAR DISORDERS | Y13098 |
| GLAUCOMA ASS WITH TUMORS OR CYSTS | Y13099 |
| GLAUCOMA+OCULAR TRAUMA | Y13100 |
| GLAUCOMA+++ EPISCLERAL VENOUS PRESSURE | Y13102 |
| HYPERSECRETION GLAUCOMA | Y13103 |
| GLAUCOMA UNS | Y13104 |
| CATARACT SEC TO GLAUCOMA :(CATARACT SECONDARY TO GLAUCOMA) | Y13127 |
| HYDROPHTHALMOS | Y17618 |
| DIABETIC GLAUCOMA | Y21847 |
| GLAUCOMA - PLATEAU IRIS | Y21855 |
| GLAUCOMA IRIDOCORNEAL ENDOTHELIAL (ICE) SYNDROME | Y21856 |
| GLAUCOMA-POSNER SCHLOSMAN SYNDROME | Y21857 |
| WEGENER'S SYND | Y14353 |
| VISUAL LOSS LEGAL BLINDNESS | 369.4 |
| VISUAL CORTEX BLINDNESS | 377.75 |
| CONG BLINDNESS | Y13244 |
| VISUAL LOSS LEGAL BLINDNESS | Y13250 |
| VISUAL CORTEX BLINDNESS | Y13587 |
| HYPOTONY EYE UNS | Y21848 |
| EYEBALL AND ORBIT BIOPSY | Y22327 |
| EYEBALL ENUCLEATION | Y22328 |
| ORBITAL WOUND REPAIR | Y22332 |
| EYEBALL RUPTURE REPAIR | Y22333 |
| ORBITAL LESION EXCISION | Y22334 |
| POST SEGMENT EYE FOREIGN BODY REMOVAL | Y22337 |
| RETINAL LESION DESTRUCTION | Y22338 |
| RETINAL TEAR REPAIR | Y22339 |
| REPAIR RETINAL DETACHMENT | Y22340 |
| VITREOUS SUBSTITUTE INJECTION | Y22342 |
| SWOLLEN OPTIC DISC | Y23375 |
| OPTIC NERVE GLIOMA | Y23775 |
| DIABETIC OPTIC NEUROPATHY | Y23950 |
| OPTIC DISC PIT | Y23955 |
| NEUROMYELITIS OPTICA | 341.0 |
| ORBITAL DIS UNS (DISORDER) | 376.9 |
| FOSTER-KENNEDY SYND | 377.04 |
| OPTIC ATROPHY | 377.1 |
| OPTIC ATROPHY PRIMARY | 377.11 |
| OPTIC ATROPHY POST INFLAMMATORY | 377.12 |
| OPTIC ATROPHY PARTIAL | 377.15 |
| OPTIC ATROPHY HEREDITARY | 377.16 |
| CRATER-LIKE HOLES OPTIC DISC | 377.22 |
| COLOBOMA OPTIC DISC | 377.23 |
| PSEUDOPAPILLEDEMA | 377.24 |
| OPTIC NEURITIS | 377.3 |
| OPTIC PAPILLITIS | 377.31 |
| RETROBULBAR NEURITIS ACUTE | 377.32 |
| NUTRITIONAL OPTIC NEUROPATHY | 377.33 |
| TOXIC OPTIC NEUROPATHY | 377.34 |
| DIABETIC OPTIC NEUROPATHY | 377.39 |
| ISCHEMIC OPTIC NEUROPATHY | 377.41 |
| HEMORRHAGE IN OPTIC NERVE SHEATHS | 377.42 |
| COMPRESSION OPTIC NERVE | 377.49 |
| OPTIC CHIASM DISS | 377.5 |
| OPTIC CHIASM DIS PITUITARY DIS | 377.51 |
| OPTIC CHIASM DISS NEOPLASTIC NOT PITUIT | 377.52 |
| OPTIC CHIASM DISS VASCULAR | 377.53 |
| OPTIC CHIASM DISS INFLAMMATORY | 377.54 |
| VISUAL PATHWAY NEOPLASM | 377.61 |
| VISUAL PATHWAY VASCULAR DIS | 377.62 |
| VISUAL PATHWAY DISS INFLAMMATORY | 377.63 |
| VISUAL CORTEX DIS | 377.7 |
| VISUAL CORTEX NEOPLASM | 377.71 |
| VISUAL CORTEX VASCULAR DIS | 377.72 |
| VISUAL CORTEX INFLAMMATORY DIS | 377.73 |
| VISUAL CORTEX BLINDNESS | 377.75 |
| OPTIC NERVE & VISUAL PATHWAYS DIS UNS | 377.9 |
| VITREOUS DEGENERATION | 379.21 |
| VITREOUS MEMBRANES & STRANDS | 379.25 |

*Y codes are MHS-specific diagnostic codes.
