# Supplementary material for: Association between eye disorders and the development of ADHD/ADD: a nationwide retrospective cohort study
Source: Eye (Lond). 2026 Jan 9;40(4):550–6. doi: 10.1038/s41433-025-04227-w (PMC12957306; doi:10.1038/s41433-025-04227-w)
Supplement: Supplementary file 4 — Supplementary Table 3 [file 41433_2025_4227_MOESM4_ESM.docx]

Supplemental Table 3. Comparison of association between general eye diagnosis and ADHD/ADD: Diagnosis alone, diagnosis with medication prescription and diagnosis with medication dispensation.

|  | No ADHD/ADD | | ADHD/ADD | | HR [95%CI] | p-value |
| --- | --- | --- | --- | --- | --- | --- |
|  | N | % | N | % |  |  |
| **ADHD/ADD** |  |  |  |  |  |  |
| Without eye diagnosis (n=443,414) | 402,386 | 67.5 | 41,028 | 59.5 | 1.40 [1.38-1.42] | <0.001 |
| With eye diagnosis (n=221,707) | 193,759 | 32.5 | 27,948 | 40.5 |  |  |
| **ADHD/ADD + Medication prescription** |  |  |  |  |  |  |
| Without eye diagnosis (n=435,324) | 402,386 | 67.5 | 32,938 | 59.5 | 1.41 [1.38-1.43] | <0.001 |
| With eye diagnosis (n=216,213) | 193,759 | 32.5 | 22,454 | 40.5 |  |  |
| **ADHD/ADD + Medication dispensation** |  |  |  |  |  |  |
| Without eye diagnosis (n=432,838) | 402,386 | 67.5 | 30,452 | 59.3 | 1.42 [1.39-1.44] | <0.001 |
| With eye diagnosis (n=214,661) | 193,759 | 32.5 | 20,902 | 40.7 |  |  |

*n represents the number of matched cases and controls (in a 1:2 ratio) with any eye diagnosis and without an eye diagnosis, respectively.

**N represents the number of participants in each cell defined by ADHD/ADD status and eye diagnosis category; % indicates the proportion within each ADHD/ADD group.
