# Supplementary material for: Association between eye disorders and the development of ADHD/ADD: a nationwide retrospective cohort study
Source: Eye (Lond). 2026 Jan 9;40(4):550–6. doi: 10.1038/s41433-025-04227-w (PMC12957306; doi:10.1038/s41433-025-04227-w)
Supplement: Supplementary file 5 — Supplementary Table 4 [file 41433_2025_4227_MOESM5_ESM.docx]

Supplemental Table 4. Associations between eye disorders and ADHD/ADD, adjusted to SES.

|  | No ADHD/ADD | | ADHD/ADD | | HR [95%CI] | p-value |
| --- | --- | --- | --- | --- | --- | --- |
|  | N | % | N | % |  |  |
| **General eye diagnosis** |  |  |  |  |  |  |
| Without eye diagnosis (n=443,414) | 402,386 | 67.5 | 41,028 | 59.5 | 1.39 [1.37-1.41] | <0.001 |
| With the diagnosis (n=221,707) | 193,759 | 32.5 | 27,948 | 40.5 |  |  |
| **Strabismus** |  |  |  |  |  |  |
| Without eye diagnosis (n=8,950) | 8,008 | 68.1 | 942 | 56.3 | 1.66 [1.51-1.83] | <0.001 |
| With the diagnosis (n=4,475) | 3,743 | 31.9 | 732 | 43.7 |  |  |
| **Amblyopia** |  |  |  |  |  |  |
| Without eye diagnosis (n=9,560) | 8,508 | 67.7 | 1,052 | 59.6 | 1.40 [1.27-1.54] | <0.001 |
| With the diagnosis (n=4,780) | 4,068 | 32.3 | 712 | 40.4 |  |  |
| **Myopia** |  |  |  |  |  |  |
| Without eye diagnosis (n=286,282) | 262,582 | 67.2 | 23,700 | 61.0 | 1.32 [1.29-1.35] | <0.001 |
| With the diagnosis (n=143,141) | 127,966 | 32.8 | 15,175 | 39.0 |  |  |
| **Hyperopia** |  |  |  |  |  |  |
| Without eye diagnosis (n=81,210) | 71,179 | 68.1 | 10,031 | 57.9 | 1.47 [1.43-1.52] | <0.001 |
| With the diagnosis (n=40,605) | 33,313 | 31.9 | 7,292 | 42.1 |  |  |
| **Astigmatism** |  |  |  |  |  |  |
| Without eye diagnosis (n=118,814) | 106,823 | 67.8 | 11,991 | 57.7 | 1.47 [1.43-1.51] | <0.001 |
| With the diagnosis (n=59,407) | 50,631 | 32.2 | 8,776 | 42.3 |  |  |

*n represents the number of matched cases and controls (in a 1:2 ratio) with the eye diagnosis in question and without an eye diagnosis, respectively.

**N represents the number of participants in each cell defined by ADHD/ADD status and eye diagnosis category; % indicates the proportion within each ADHD/ADD group.

***SES – Socioeconomic status.
