# Supplementary material for: Association between eye disorders and the development of ADHD/ADD: a nationwide retrospective cohort study
Source: Eye (Lond). 2026 Jan 9;40(4):550–6. doi: 10.1038/s41433-025-04227-w (PMC12957306; doi:10.1038/s41433-025-04227-w)
Supplement: Supplementary file 6 — Supplementary Table 5 [file 41433_2025_4227_MOESM6_ESM.docx]

|  | Without eye diagnosis  Mean ± SD, median [Q1-Q3] | With the diagnosis  Mean ± SD, median [Q1-Q3] | p-value |
| --- | --- | --- | --- |
| **General eye disorder** | | | |
| N | 41,028 | 27,948 |  |
| Time to ADHD/ADD diagnosis | 4.9±2.9, 4.2 [2.4-7.0] | 4.5±2.8, 3.8 [2.2-6.4] | <0.001 |
| **Strabismus** | | | |
| N | 942 | 732 |  |
| Time to ADHD/ADD diagnosis | 4.9±2.9, 4.3 [2.6-7.0] | 4.2±2.8, 3.3 [2.0-5.9] | <0.001 |
| **Amblyopia** | | | |
| N | 1,052 | 712 |  |
| Time to ADHD/ADD diagnosis | 4.6±2.9, 3.8 [2.2-6.5] | 4.3±2.8, 3.5 [2.1-6.1] | 0.080 |
| **Myopia** | | | |
| N | 23,700 | 15,175 |  |
| Time to ADHD/ADD diagnosis | 5.1±3.0, 4.5 [2.5-7.3] | 4.7±2.9, 4.0 [2.3-6.7] | <0.001 |
| **Hyperopia** | | | |
| N | 10,031 | 7,292 | <0.001 |
| Time to ADHD/ADD diagnosis | 4.4±2.7, 3.7 [2.2-6.1] | 4.2±2.7, 3.5 [2.1-5.8] |  |
| **Astigmatism** | | | |
| N | 11,991 | 8,776 | <0.001 |
| Time to ADHD/ADD diagnosis | 4.8±2.9, 4.2 [2.3-6.9] | 4.5±2.8, 3.7 [2.1-6.4] |  |

Supplemental Table 5. Time to ADHD/ADD diagnosis, by presence of an eye disorder.
