# Supplementary material for: Association between eye disorders and the development of ADHD/ADD: a nationwide retrospective cohort study
Source: Eye (Lond). 2026 Jan 9;40(4):550–6. doi: 10.1038/s41433-025-04227-w (PMC12957306; doi:10.1038/s41433-025-04227-w)
Supplement: Supplementary file 7 — Supplementary Table 6 [file 41433_2025_4227_MOESM7_ESM.docx]

Supplemental Table 6. Associations between general eye disorder and ADHD/ADD, by age- and sex- groups.

| Age groups | 5-18 years | | | | | | 18-30 years | | | | | | p-value ^1^ |
| --- | --- | --- | --- | --- | --- | --- | --- | --- | --- | --- | --- | --- | --- |
|  | No ADHD/ADD | | ADHD/ADD | | HR [95%CI] | p-value | No ADHD/ADD | | ADHD/ADD | | HR [95%CI] | p-value |  |
|  | N | % | N | % |  |  | N | % | N | % |  |  |  |
| Without eye diagnosis (n=443,414) | 268,379 | 67.7 | 34,127 | 59.4 | 1.42 [1.39-1.44] | <0.001 | 134,007 | 67.1 | 6,901 | 59.8 | 1.37 [1.32-1.42] | <0.001 | 0.044 |
| With eye diagnosis (n=221,707) | 127,944 | 32.3 | 23,309 | 40.6 |  |  | 65,815 | 32.9 | 4,639 | 40.2 |  |  |  |
|  | | | | | | | | | | | | | |
| Sex groups | Male | | | | | | Female | | | | | | p-value ^1^ |
|  | No ADHD/ADD | | ADHD/ADD | | HR [95%CI] | p-value | No ADHD/ADD | | ADHD/ADD | | HR [95%CI] | p-value |  |
|  |  |  |  |  |  |  |  |  |  |  |  |  |  |
| Without eye diagnosis (n=443,414) | 162,636 | 67.5 | 19,534 | 60.4 | 1.35 [1.32-1.38] | <0.001 | 239,750 | 67.5 | 21,494 | 58.6 | 1.45 [1.42-1.48] | <0.001 | <0.001 |
| With eye diagnosis (n=221,707) | 78,293 | 32.5 | 12,792 | 39.6 |  |  | 115,466 | 32.5 | 15,156 | 41.4 |  |  |  |

*n represents the number of matched cases and controls (in a 1:2 ratio) with the eye diagnosis in question and without an eye diagnosis, respectively.

**N represents the number of participants in each cell defined by ADHD/ADD status and eye diagnosis category; % indicates the proportion within each ADHD/ADD group.

^1^Test for heterogeneity
